# ASSOCIATION OF COGNITIVE STATUS AND CONSUMPTION OF UNPROCESSED AND ULTRA-PROCESSED FOODS IN BRAZILIAN OLDER ADULTS

**DOI:** 10.1093/geroni/igac059.2957

**Published:** 2022-12-20

**Authors:** Graziele Silva, Carol Freiria, Tábatta Brito, Flávia Arbex Silva Borim, Ligiana P Corona

**Affiliations:** UNICAMP, Limeira, Sao Paulo, Brazil; Universidade Estadual de Campinas, Campinas, Sao Paulo, Brazil; Universidade Federal de Alfenas, Alfenas, Sao Paulo, Brazil; Universidade Estadual de Campinas, Campinas, Sao Paulo, Brazil; Universidade Estadual de Campinas/ Faculdade de Ciências Aplicadas, Limeira, Sao Paulo, Brazil

## Abstract

The decrease in consumption of fresh or minimally processed foods and the increase in ultra-processed foods are being observed in the diet of older adults and these changes may lead to worsening health status and cognition. We aimed to evaluate the association between cognitive status and food consumption according to the level of processing in Brazilian older adults. Cross-sectional study, with a sample of 585 older adults (≥60 years). Cognition was evaluated using the Cognitive Skills Screening Instrument (CASI-S), considering cognitive deficit when scores < 23 in participants aged 60–69 and < 20 in those aged ≥70 years. Foods reported in 24-hour food recall were classified according to their processing level into four groups of NOVA proposal: 1) unprocessed/minimally processed foods, 2) culinary ingredients, 3) processed foods (products made only from groups 1 and 2); and 4) ultra-processed foods. We estimated the means of total CASI-S score and its four domains according to the quartiles of intake of each food group, and evaluated the association between cognitive decline and each food group intake using logistic models adjusted for gender, age, schooling. Individuals in the highest quartile of unprocessed/minimally processed foods intake had higher scores in temporal orientation (p=0.034), verbal fluency (p=0.002), and total CASI-S score (p=0.004). The scores did differ according to the intake of the other food groups. The ultra-processed was the only group associated with cognitive deficit (OR:1.02; p=0.002). Results suggest nutritional counselling for older adults should focus in reducing ultra-processed and increasing unprocessed foods to help preventing cognitive deficit.

